# Classification of Pulmonary Nodules in 2-[^18^F]FDG PET/CT Images with a 3D Convolutional Neural Network

**DOI:** 10.1007/s13139-023-00821-6

**Published:** 2023-08-30

**Authors:** Victor Manuel Alves, Jaime dos Santos Cardoso, João Gama

**Affiliations:** 1 Faculty of Economics, University of Porto, Rua Dr. Roberto Frias, Porto, 4200-464 Porto, Portugal; 2 Department of Nuclear Medicine, University Hospital Center of São João, Alameda Prof. Hernâni Monteiro, 4200-319 Porto, Portugal; 3 Faculty of Engineering, University of Porto, Rua Dr. Roberto Frias, 4200-465 Porto, Portugal; 4 Institute for Systems and Computer Engineering, Technology and Science (INESC TEC), Rua Dr. Roberto Frias, 4200-465 Porto, Portugal

**Keywords:** Convolutional neural networks, Positron emission tomography, 2-[^18^F]FDG PET/CT, Pulmonary nodules, Artificial intelligence

## Abstract

**Purpose:**

2-[^18^F]FDG PET/CT plays an important role in the management of pulmonary nodules. Convolutional neural networks (CNNs) automatically learn features from images and have the potential to improve the discrimination between malignant and benign pulmonary nodules. The purpose of this study was to develop and validate a CNN model for classification of pulmonary nodules from 2-[^18^F]FDG PET images.

**Methods:**

One hundred thirteen participants were retrospectively selected. One nodule per participant. The 2-[^18^F]FDG PET images were preprocessed and annotated with the reference standard. The deep learning experiment entailed random data splitting in five sets. A test set was held out for evaluation of the final model. Four-fold cross-validation was performed from the remaining sets for training and evaluating a set of candidate models and for selecting the final model. Models of three types of 3D CNNs architectures were trained from random weight initialization (Stacked 3D CNN, VGG-like and Inception-v2-like models) both in original and augmented datasets. Transfer learning, from ImageNet with ResNet-50, was also used.

**Results:**

The final model (Stacked 3D CNN model) obtained an area under the ROC curve of 0.8385 (95% CI: 0.6455–1.0000) in the test set. The model had a sensibility of 80.00%, a specificity of 69.23% and an accuracy of 73.91%, in the test set, for an optimised decision threshold that assigns a higher cost to false negatives.

**Conclusion:**

A 3D CNN model was effective at distinguishing benign from malignant pulmonary nodules in 2-[^18^F]FDG PET images.

**Supplementary Information:**

The online version contains supplementary material available at 10.1007/s13139-023-00821-6.

## Introduction

Lung cancer is the leading cause of cancer death worldwide [1]. The prognosis is strongly dependent on the tumour stage at the diagnosis time [2]. The early diagnosis and treatment of lung cancer is essential for reducing the mortality of this type of cancer [3].

In the early stages, lung cancer is usually asymptomatic and often presents as a pulmonary nodule [2]. However, pulmonary nodules may have several causes [4]. They are a common incidental finding on imaging scans performed for various indications [4]. They are also a common finding on scans performed for screening of lung cancer [5]. In both cases, most nodules are benign, but a small proportion represent lung cancer, usually at an early stage; hence it is important to identify them correctly [5, 6].

The management of pulmonary nodules requires an initial evaluation of malignancy risk by a computed tomography (CT) scan of the thorax [7, 8]. The subsequent diagnostic work-up may include a 2-deoxy-2-[^18^F]fluoro-D-glucose (2-[^18^F]FDG) positron emission tomography/computed tomography (PET/CT) for metabolic characterisation of solid or partially solid pulmonary nodules [7, 8]. The British Thoracic Society guidelines [8] recommend a 2-[^18^F]FDG PET/CT for solid nodules with ≥10 mm in diameter and a malignancy risk >10%. The Fleischner Society guidelines [7] consider 2-[^18^F]FDG PET/CT as an option for evaluation of solid nodules with >8 mm in diameter and partially solid nodules with a solid component >8 mm in diameter.

The inclusion of 2-[^18^F]FDG PET/CT in the diagnostic work-up reduces the proportion of futile invasive diagnosis procedures [8], which can be a source of complications [4]. On the other hand, a PET/CT would anticipate a potential diagnosis of lung cancer compared to a CT surveillance strategy.

2-[^18^F]FDG PET/CT interpretation relies on intensity of tracer uptake in the lesion (nodule-to-background contrast) through qualitative [9] or quantitative analysis [10]. Classical machine learning models for supporting the differential diagnosis of pulmonary nodules have been developed from PET imaging features (SUVmax and/or radiomics), in some cases, also combined with either CT imaging features (radiomic or visually extracted features) or non-imaging features [9, 11–20]. Training machine learning models with radiomic features requires the extraction of dozens of handcrafted features and a laborious process of feature selection [21, 22]. In addition, the radiomic features are sensitive to variations in the image acquisition and reconstruction, segmentation, image processing and feature computation in multi-center setting, and extensive standardization and harmonisation are required to obtain reproducible models [21–23].

Deep learning is a subfield of machine learning, which in turn is part of the artificial intelligence. Deep learning models can learn useful representations for the predictive task, directly from labelled raw data [24], such as images, having the potential to improve the classification of pulmonary nodules.

Deep learning models have been successful in medical imaging. They have reached comparable performance to physicians or even outperformed them in specific tasks, in areas as diverse as dermatology [25–27], ophthalmology [28–31], pathological anatomy [32–34] or radiology [35–38].

The main objective of this research was to develop a convolutional neural network (CNN) model for classification of pulmonary nodules from an annotated dataset of 2-[^18^F]FDG positron emission tomography (PET) images. Secondarily, the hypothesis that the model outperforms the maximum standardised uptake value (SUVmax) was tested. Explanations for the decisions of the model were obtained by gradient-weighted class activation mapping (Grad-CAM).

## Materials and Methods

This study was conducted in accordance with the Declaration of Helsinki and national regulations. The study was approved by the University Hospital Centre of São João, Porto, Portugal, which included approval by the institutional Ethics Committee and the Responsible for Data Reuse. The informed consent of the participants was waived due to the retrospective nature of the research.

### Image Dataset

A PET image dataset of pulmonary nodules was created. To ensure the quality of the data for modelling, the eligible population, the reference standard and the sampling procedure were first determined. Then, the data were collected and preprocessed.

#### Eligible Population

The participants belong to the eligible population if they cumulatively meet the following inclusion criteria:

- One or more indeterminate solid pulmonary nodules with more than 8 mm in average diameter. The average diameter should not exceed the 30 mm, according to the nodule definition provided by the British Thoracic Society guidelines [8]. The average diameter of the nodule corresponds to the average of long-axis and perpendicular short-axis diameters, both of which are obtained on the same orthogonal slice, such as defined in the Fleischner Society Guidelines [7];
- The nodule detection was incidental or through screening;
- 2-[^18^F]FDG PET/CT was performed for clarification of the nodule(s), and the reconstructed images are available in digital format. The pathological status of the nodule(s) is unknown at the time of the PET/CT (indeterminate nodule);
- The nodule was biopsied or excised and obtained a histopathological or cytopathological examination, otherwise completed an imaging follow-up period.

Those with at least one of the following criteria were excluded:

- History of lung cancer;
- History of other cancers, except:
- Non-melanoma skin cancer, low-risk localised prostate cancer, in situ cervical cancer, in situ breast cancer, or superficial bladder cancer, which has been treated at least 6 months ago.

#### Reference Standard

The reference standard for the pulmonary nodule status was defined on the basis of the histopathological or the cytopathological examination, and/or the nodule behaviour during the follow-up period with CT. It attributes one of two classes (benign or malignant) to the target feature which is the status of each pulmonary nodule as following:

1. A nodule is defined as malignant if biopsied or excised during the initial diagnostic workup or during the follow-up period, and the histopathological or cytopathological examination shows a malignant neoplasm.
2. A nodule is defined as benign if:
  - Excised and the histopathological examination showed benign pathology;
  - Biopsied, the biopsy was diagnostic and the histopathological examination showed a benign pathology;
  - Neither excised nor biopsied, or biopsied but non-diagnostic and during follow-up:
    - The nodule disappeared;
    - The nodule decreased or kept the same size for, at least, 2-year of follow-up;
    - The nodule increased in size and thereafter was biopsied or excised and the histology was benign;
    - Volume doubling time >600 days and <25% change in volume for, at least, 1 year of follow-up.

A minimum of 2-year imaging follow-up was established for solid nodules when the mean axial diameter of the nodule was used for follow-up. When the follow-up period was between 1- and 2-year, the nodular volume was estimated from the diameter on three orthogonal axes. These follow-up criteria are based on the doubling time of malignant solid nodules and are recommended for pulmonary nodule management [7, 8].

#### Sampling

Every patient referred to the University Hospital Centre of São João and who underwent a 2-[^18^F]FDG PET/CT scan between 2010 and 2019 was consecutively selected if he/she belongs to the defined population.

If a patient underwent more than a PET/CT scan, only the first one was considered. If a patient has more than one nodule that fills the eligibility criteria, only the more suspicious was included.

Among the 7130 PET/CT scan requests within the established time interval, the 2-[^18^F]FDG PET/CT scans that aimed at clarifying the diagnosis of pulmonary nodules were selected. Then, the eligibility criteria were checked for those by consulting the medical records and the information of the histopathological/ cytopathological examination, the standard-dose CT scan and the 2-[^18^F]FDG PET/CT scan. In the end, 113 participants were eligible to create a PET image dataset.

#### Image Acquisition, Preprocessing, and Annotation

All patients underwent a PET/CT scan with a field of view between the skull base and mid-thighs around 60 min after the 2-[^18^F]FDG intravenous injection. The exams were acquired in three different scanners (GE Discovery IQ 4R, GE Discovery LS/4 and Siemens Biograph 6). The PET images were reconstructed using the ordered subset expectation maximisation method. Attenuation correction of PET data was performed with low-dose CT-derived attenuation maps.

The image preprocessing was performed on 3D Slicer 4.10.2 r28257 [39]. Both the PET and CT image files were imported and coregistered with rigid registration. Once the PET/CT scans were performed in different scanners, the PET volumes have different voxel size and anisotropic spacing. Therefore, the volumes were resampled to obtain the same voxel size and isotropic voxels. The voxel side was set to 1.5 mm which is a smaller size than the smaller voxel side of the three scanners. Linear interpolation was used for spatial resampling.

The nodule was visually identified in the coregistered PET/CT images, and a cubic region of interest was drawn and cropped to include the entire nodule. The center of this subvolume coincides with the center of the nodule. The subvolume of interest has a side length equal to twice the maximum possible diameter of the nodule (60 mm × 60 mm × 60 mm). The obtained subvolume was saved in .nrrd format. Each cropped subvolume containing a pulmonary nodule was annotated with the corresponding class of the target feature (benign or malignant).

### Formulation of the Deep Learning Task

The supervised deep learning problem is a single task, single label, binary classification problem that inputs cubic regions of interest from PET for a three-dimensional (3D) CNN.

Let X be a random variable that represents an input, i.e. a PET image, which corresponds to a tensor, being the axes 1, 2 and 3, the shape of the volume-of-interest (40 × 40 × 40) and the axis 4, the number of channels, in this case only one. Let Y be a random variable which corresponds to the target. Let S be a training set with *n* pairs (*X*, *Y*) of independent and identically distributed samples drawn from the population. Then, the learning problem consists of using a CNN-based algorithm for choosing from the hypothesis space, the hypothesis or model that best approximates an unknown mapping function f: *X*→*Y* in the population, using the training set as a starting point. Model training is performed by discovering the parameter configuration that minimises a loss function in the training set, the structural risk, a surrogate of the expected risk [40, 41]. However, minimising the risk in the training set is prone to overfitting and a dissociation between the expected and structural risks occurs at any time during the training [42]. An estimate of expected risk in the validation set is more accurate, but cannot be used to update model parameters, so it may be used to decide when to stop training [42].

### Experimental Setup

#### Input Data Splitting

The dataset was randomly split into five stratified partitions of similar size. The stratification was performed by the target class in order to maintain the same class distribution of the original data in each data partition. Four partitions were used for 4-fold cross-validation, and the fifth one was reserved for testing. In each fold of cross-validation, three out of four partitions were used for training, and the remaining one was for model evaluation. Therefore, 4-fold cross-validation was used for training, evaluating, hyperparameter tuning and comparing different models that were built from different network architectures and, in the end, for choosing the best model. Cross-validation was preferred because it guarantees lower variance than the holdout method for the size of the obtained dataset [43].

Since tuning a model is a repetitive process, there is some leakage of information from the validation partition into the model, even it is not directly trained on it, resulting in overfitting of the model to the validation set and optimistic performance metrics [44]. For obtaining of unbiased estimates of the model performance, a test set partition was used only once to evaluate the best model, which was selected among all those trained during the cross-validation phase.

The input data for the network were subjected to fold-specific min–max normalisation to the range [0, 1]. The validation and test sets were also normalised with values of the training set of the respective fold. Data were randomly shuffled on every epoch during the training.

**Fig. 1:**
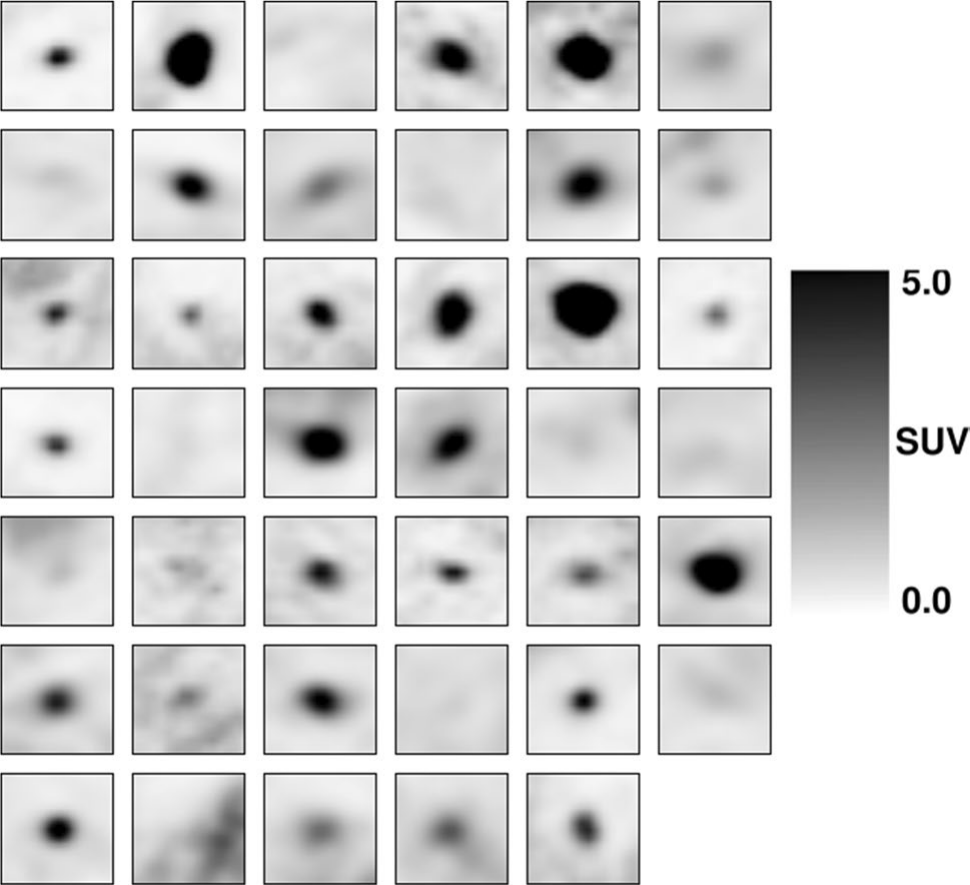
Cross-validation dataset. Middle axial slice of each PET volume. Malignant pulmonary nodules

**Fig. 2:**
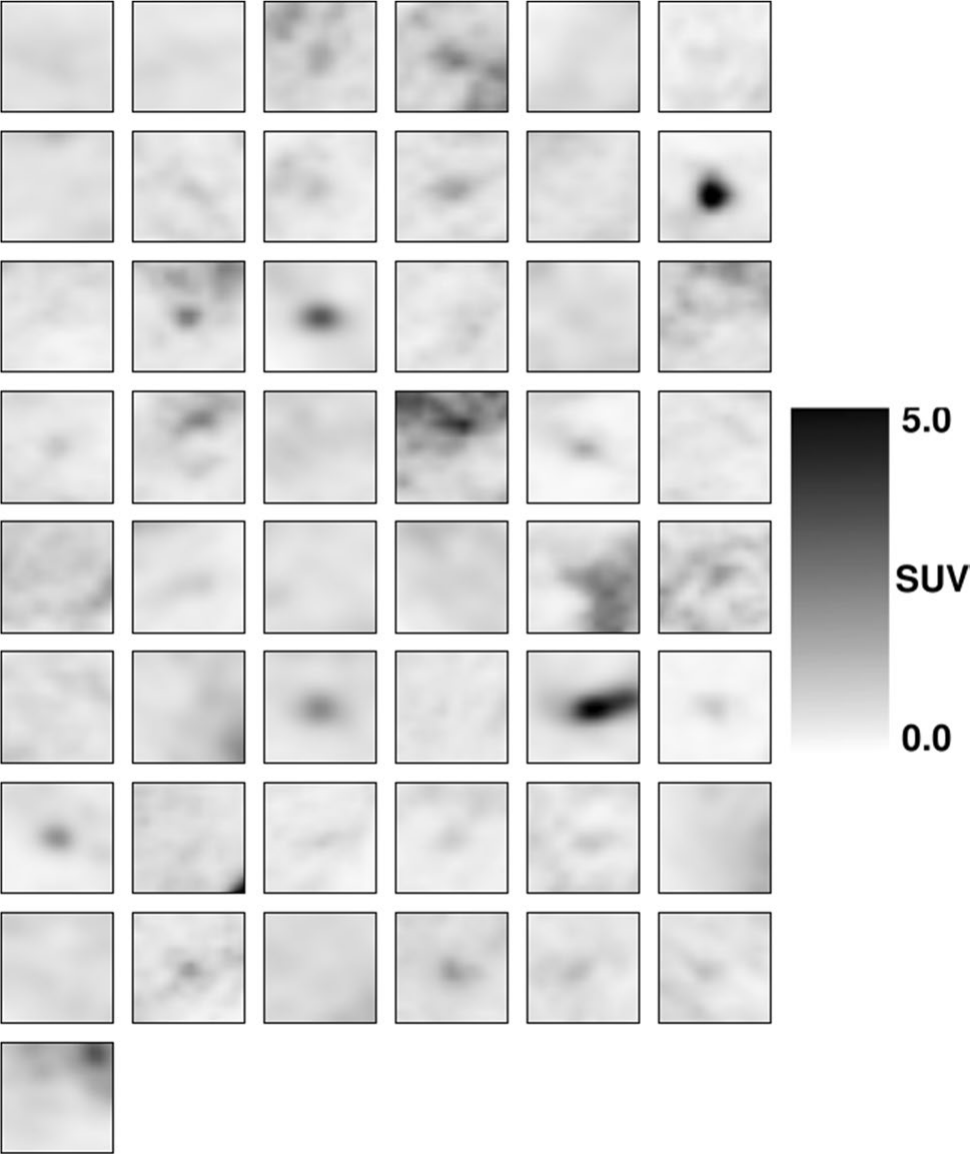
Cross-validation dataset. Middle axial slice of each PET volume. Benign pulmonary nodules

Figures 1 and 2 represent the middle axial slice of each PET volume that composes the cross-validation dataset grouped by the target class. The test set was not represented to avoid information leakage during the construction of the models.

#### Data Augmentation

Offline data augmentation [45] was performed independently in each of the four partitions of the original dataset previously created for cross-validation. Translations, rotations and Gaussian noise injection were separately applied to the original images. The test set was not augmented. The augmentation factor for each type of operation was class-specific in order to perform class balancing (Table 1). The dataset comprises original and augmented images, having around 4900 images. The size of the augmented dataset was determined by the computational resources available for training the models in a larger dataset. During the cross-validation, the models were trained in an augmented training set on each fold. The evaluation occurred in the corresponding validation set of the original dataset.

**Table 1:** Data augmentation factor

Transformation	Minority class^a^	Majority class
Translations	20	16
Rotations	21 (7 by axis)	15 (5 by axis)
Noise injection^b^	20	16

^a^The positive class (malignant nodule) is the minority class

^b^Augmentation factor was 7, 7 and 6 for the minority class and 6, 5 and 5 for the majority class, for a standard deviation of 0.1, 0.3 and 0.5, respectively

Translations were random shifts between −10 and 10 pixels on any of the 3 axes of each original image. A maximum amplitude of 10 pixels (15 mm) was chosen to ensure that the nodules were not moved out of the tensor and the label was preserved.

Random rotations between −45 and 45° were separately applied around the *x*-, *y*- or *z*-axis of each image so that each one yields augmented examples with different rotation axes, but each new augmented example has a rotation applied only around a given axis. Since the rotations were around an axis which runs through the centre of the tensor, a rotation was actually a composite operation (*P*_rotated_ = *T*^−1^ × *R* × *T* × *P*), where *P* is the voxel, *T* a translation operation and *R* a rotation operation [46]. After the spatial transformation of the coordinates of the voxels, an intensity interpolation with a bilinear interpolator was applied.

Gaussian noise with a mean of zero and three different values of standard deviation (0.1, 0.3 and 0.5) was added to the original images. The reason for adding Gaussian noise was to be able to model the PET image noise [47], so that different augmented images simulate PET images with different levels of noise.

The background voxels were filled with zero in all the above operations.

#### Training Procedures

The experiment was run in R language [48]. R Interfaces for Tensorflow (v. 2.2.0) [49] and Keras (v. 2.3.0.0) [50] and r-reticulate package [51] along with Tensorflow 2.1.0 [52] and Python 3.7.8 [53] were employed. The graphic card used was an NVIDIA GeForce MX150.

Models were trained with binary cross-entropy loss [41] and Adam optimiser [54] throughout the entire experiment. The learning rate was tuned until the optimal value was reached. The learning rate of the different models is shown in the Table 2.

**Table 2:** Models trained by cross-validation

Type	Batch size	Architecture	Learning rate	Regularizer
Stacked 3D CNN	68	conv(8,3,3,3) + mpool + conv(16,3,3,3) + mpool + conv(32,3,3,3) + mpool + conv(64,3,3,3) + flatten + fcn(32,16,1)	0.001	L2(0.00098)
Stacked 3D CNN	8	conv(8,3,3,3) + mpool + conv(16,3,3,3) + mpool + conv(32,3,3,3) + mpool + conv(64,3,3,3) + flatten + fcn(32,16,1)	0.0001	L2(0.03) and data augmentation
VGG-like	68	conv(8,3,3,3) + overlap mpool + conv(16,3,3,3) + conv(16,3,3,3) + overlap mpool + conv(32,3,3,3) + conv(32,3,3,3) + conv(32,3,3,3) + overlap mpool + flatten + fcn(1)	0.0005	L2(0.002)
VGG-like	8	conv(8,3,3,3) + overlap mpool + conv(16,3,3,3) + conv(16,3,3,3) + overlap mpool + conv(32,3,3,3) + conv(32,3,3,3) + conv(32,3,3,3) + overlap mpool + flatten + fcn(1)	0.0001	L2(0.06) and data augmentation
Inception-v2-like	16	conv(8,3,3,3) + mpool + Inception + Inception + Reduction + Inception + Inception + Reduction + gap + fcn(1)	0.0005	L2(0.0006)
Inception-v2-like	8	conv(8,3,3,3) + mpool + Inception + Inception + Reduction + Inception + Inception + Reduction + gap + fcn(1)	0.0001	L2(0.04) and data augmentation
ResNet-50 pre-trained	68	ResNet-50 (base) + gap + fcn (8,1)	5 × 10^−7^	Transfer learning and dropout(0.5)

The stopping criterion of the training corresponds to the minimum validation loss with a patience of ten or a maximum of 100 epochs. The model derived from the training epoch with the lowest validation loss was saved. This procedure was repeated for each fold of the 4-fold cross-validation, resulting four model versions, which have different values of parameters, but identical hyperparameter configuration. Early stopping ensures that the minimisation of the structural risk does not occur beyond the point of the best generalisation, obtaining a regularising effect [55].

The original dataset was trained with full-batch learning or with a mini-batch learning with batch size of 16, according to the type of network. Mini-batch learning with batch size of eight was preferred with augmented data.

Other specifications of the training procedures were changed according to the network architecture or even in networks of the same architecture (i.e. treated as hyperparameters), being explained in more detail in the next section.

### Network Architecture

Three types of 3D CNN architectures with volumetric inputs were defined. These networks were generalised from the homologous 2D CNNs (Alexnet [56], VGGNet [57] and Inception-v2 [58]), and the size of the networks was adapted to the complexity of the problem and the size of the dataset. As such, number and arrangement of layers, number of filters, kernel size and other network specifications were treated as hyperparameters, which were tuned until the proposed models were found. These networks were trained using either the original or the augmented datasets. Additionally, a 2D pre-trained model was fined-tuned in the original dataset. Some details of the different network architectures are in the Table 2.

Leaky ReLU (with *α* = 0.3) was the preferred activation function in 3D CNN because of allowing a small, non-zero gradient when a unit is not active and thus prevents ‘dying ReLU’ [59, 60].

Weights were randomly initialised according to the scheme proposed by He et al. [61], which was specifically developed to address the rectifiers.

**Fig. 3:**
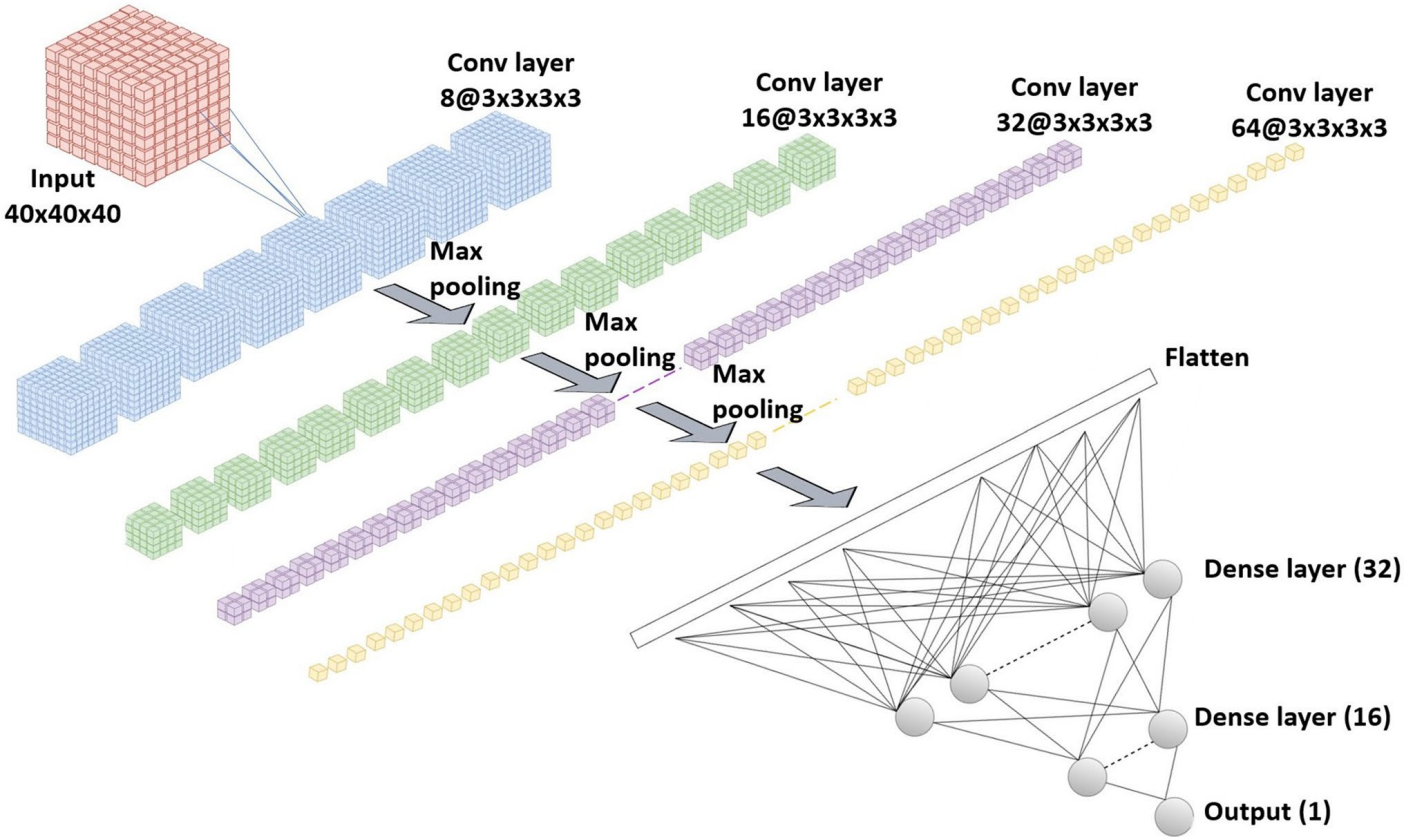
Architecture of the Stacked 3D CNN network (final model)

A network architecture inspired by Alexnet [56] was proposed. Named as Stacked 3D CNN, it is characterised by four 3D convolutional layers and three 3D max-pooling layers alternately stacked and connected to three fully connected layers (32, 16 and one units, respectively). The first convolutional layer has eight filters. Network width increases along the convolutional base by doubling the number of filters every convolutional layer. The kernel size was 3 × 3 × 3, and the kernel stride was one in the convolutional layers. No padding was applied. Pooling layers consist of max-pooling operations with kernel size of 2 × 2 × 2, stride of two and no padding. The first fully connected layer receives the output of the last convolutional layer after being flattened into a vector (Fig. 3).

The VGG-like network is characterised by a total of ten layers, being multiple stacked convolutional layers, some of them followed by a pooling layer. The output of the last convolutional layer is flattened before a fully connected output layer (Figure A1 of Supplementary Information). The efficient use of 3 × 3 × 3 convolutions is a prominent property of this type of network [57]. Thus, convolutions of larger kernel size are factorised into 3 × 3 × 3, while the receptive field is preserved. Consequently, the depth of the network increases, while the number of parameters is reduced. More specifically, 5 × 5 × 5 and 7 × 7 × 7 convolutional layers are replaced by sets of two or three 3 × 3 × 3 convolutional layers. Factorisation of convolutions imposes a greater reduction in the number of parameters in a 3D than in a 2D network, and therefore a greater regularising effect (Section A.1.1 of Supplementary Information). Both expansion of feature maps and decreasing of the spatial resolution only occur after each pooling layer. Overlapping max-pooling [56] with a pool size of 3 × 3 × 3, strides of two and padding was applied.

The Inception-v2-like network is a 3D CNN with three main characteristics—inception modules, 1 × 1 × 1 convolutions and factorisation of convolutions—which introduce sparsity in the network and reduce the number of parameters, making the network more efficient [58]. Inception modules consist of blocks of several convolutional layers with different kernel size and a pooling layer that receive the same input, propagate the information in parallel and concatenate the output before passing it to the next layer [58]. Much of the computational efficiency is achieved by using 1 × 1 × 1 convolutions to compute reductions of the number of feature maps before expensive convolutions of larger kernel size. Factorisation of convolutions of larger kernel size into stacks of 3 × 3 × 3 or asymmetrical convolutions while preserving the receptive field provides a further increase in efficiency. There are two types of inception modules—one standard module for learning representations and another one that simultaneously downsizes the feature maps [58]. Two versions of each were implemented in the network. The proposed network has four standard inception modules and two reduction modules. The output of the last convolutional layer is converted into a vector by global average pooling, which is received by a fully connected output layer (Figures A2 to A6 of Supplementary Information).

Transfer learning [62] from Imagenet dataset [63] was also performed. Pre-trained Resnet-50 [64] was used as a feature extractor. Two fully connected layers were added to its top and initialised according to He et al. [61]. Because the dataset of the current problem is quite different from that of the source domain, only the earlier layers of ResNet-50 were used (until conv3_block1_out). Additionally, a few of the top layers (from conv3_block1_1_conv) were fine-tuned with a very low learning rate. Due to the 2D architecture, the input for this network consists of 3 central slices (19, 20 and 21) of the PET volume, which are orthogonal to the third axis, being each one stored in a different channel.

Besides the regularisation procedures already described, L2 regularisation [42] was applied to all layers with parameters of the 3D CNN models, whereas dropout [65] was applied to the fully connected layers of the 2D CNN model.

### Performance Metrics and Model Selection

The performance metric selected to evaluate the models was the area under the receiver operating characteristic (ROC) curve. It was computed with the trapezoidal rule from non-parametric ROC curves [66]. During the cross-validation, model evaluation was conducted in the validation partition of the respective fold. Different models were compared by their mean area under the ROC curve of the 4-folds.

Models with different network architectures were trained. In order to deal with a source of non-determinism on Tensorflow GPU^1^, the best model of each network architecture was retrained and evaluated again by 10 iterations, under identical conditions, on the 4-fold cross-validation. The average performance metrics over the 10 iterations for the different models were compared, and the best model was selected. Since that model has 10 versions for each fold, one of them was randomly picked.

Subsequently, an ensemble classifier was built from the four versions of the best model derived from the 4-fold cross-validation, by averaging their output probabilities, weighted by the size of each training partition. This ensemble classifier was evaluated in the test set to determine its generalisation performance over unseen examples. The 95% confidence interval of the area under the ROC curve was also determined for the test set according to the method described by DeLong [67].

Accuracy, sensitivity and specificity were complementary metrics determined in the test set. Instead of using the standard decision threshold of 0.5, an optimal decision threshold was determined for each version of the best model in the respective validation partition. The four decision thresholds were averaged, and the resulting threshold was applied to convert the output probabilities of the ensemble model into classes, in the test set. If the predicted probability was equal to or higher than the threshold, the nodule was classified as malignant; otherwise, it was classified as benign. The optimal threshold was determined according to two different approaches. In the first one, the value of the optimal threshold was the posterior probability which maximises the Youden index [68]. In another scenario, the cost of a false negative was considered higher than the cost of a false positive. Therefore, a minimum sensitivity was set to 95%, and the cut-off point which maximises the specificity was searched.

### Paired Comparison Between the Final CNN Model and the SUVmax

As a secondary analysis, a hypothesis test was performed, in the test set, to infer about a possible difference in the area under the ROC curve between the final CNN model and the SUVmax in the population (H_1_: AUC ROC_CNN_ ≠AUC ROC_SUVmax_), starting from the null hypothesis of equality. The type I error (*α*) was predefined as 0.05.

The non-parametric test developed by DeLong et al. [67], which makes a paired comparison of the area under the ROC curves, is applied if the area of one ROC curve is uniformly higher than the other across all operating points, that is, the curves do not cross each other; otherwise, a hypothesis test based on the ROC shape proposed by Venkatraman and Begg [69] is applied.

### Model Explainability

Grad-CAM analysis [70] was applied to generate visual explanations for the decisions of the model. This method highlights the most class-discriminant regions of a volume-of-interest under the 3D CNN classification model standpoint. Insights about how the model succeeded or failed were obtained. The Grad-CAM 3D heatmap was obtained for each PET volume of the test set from each of the four 3D CNN model versions which compose the ensemble model. Fusion images were created by superimposing the axial slices of the Grad-CAM 3D heatmap and the axial slices of the original input PET volume for selected cases. The volumes were reprocessed to obtain ten axial slices rather than forty to facilitate the representation of the images. Red and dark red tones represent higher Grad-CAM score for a class, as such they were the most relevant regions of the input volume for model decision.

## Results

### Descriptive Statistics

The dataset has 113 participants. Seventy-six (67.3%) of participants were male. The median age was 65 years old (interquartile range (IQR): 14 years). One nodule was included by participant. The median diameter of the nodule in low-dose CT scan was 13 mm (IQR: 5 mm). Fifty-one (45.1%) malignant pulmonary nodules were found; the remaining were benign. Table 3 shows the distribution of the nodules according to the type, detailing the histological type of the malignant nodules.

**Table 3:** Characterisation of the pulmonary nodules according to the histological type

Class	*n* (%)
Adenocarcinoma	31 (27.4)
Squamous cell carcinoma	4 (3.5)
Small cell lung cancer	2 (1.8)
Large cell carcinoma	2 (1.8)
Carcinoid tumour	7 (6.2)
Metastasis	0 (0.0)
Other/uncertain cancer	5 (4.4)
Benign nodule	62 (54.9)

The reference standard was obtained by histological examination, cytological examination or follow-up CT scan in 71 (62.8%), 1 (0.9%) and 41 (36.3%) of the nodules, respectively. When the reference standard was obtained by follow-up CT scan, the median follow-up was 2.6 years (minimum: 1.3 years; maximum: 8.3 years), and 85.4% of the participants had a follow-up time ≥ 2 years.

### Evaluation of CNN Models by 4-Fold Cross-Validation

Table 4 shows the area under the ROC curve for the CNN models evaluated by 4-fold cross-validation. The classification performance measured by this metric ranged between 0.8864 for a Stacked 3D CNN model and 0.7738 for a ResNet-50 pre-trained model. Regardless of the type of model, it was consistently found that models trained on the original dataset performed better than those trained on the augmented dataset.

**Table 4:** Evaluation of the CNN models by 4-fold cross-validation

Area under the ROC curve						
Model	F1	F2	F3	F4	Mean	SD^a^
Stacked 3D CNN	0.7917	0.9000	0.8750	0.9790	0.8864	0.0772
Stacked 3D CNN + Augmentation	0.7333	0.8500	0.8417	0.9371	0.8405	0.0835
VGG-like	0.7333	0.9250	0.9417	0.9161	0.8790	0.0977
VGG-like + Augmentation	0.7000	0.7833	0.8667	0.9301	0.8200	0.1001
Inception-v2-like	0.7250	0.9083	0.8917	0.9650	0.8725	0.1032
Inception-v2-like + Augmentation	0.7333	0.8333	0.8417	0.8741	0.8206	0.0608
ResNet-50 pre-trained	0.7167	0.8083	0.8500	0.7203	0.7738	0.0662

^a^*SD* standard deviation

The retraining and evaluation over 10 iterations of 4-fold cross-validation has resulted in a mean area under the ROC curve of 0.8822, 0.8760 and 0.8690 for the best models of each architecture (Stacked 3D CNN, VGG-like and Inception-v2-like models, respectively), all trained in the original dataset (Tables A1 to A3 of Supplementary Information). ResNet-50 was not retrained because its performance was much lower than other architectures. The Stacked 3D CNN model showed consistently the best performance on the iterated cross-validation.

### Evaluation of the Final CNN Model in the Test Set

**Fig. 4:**
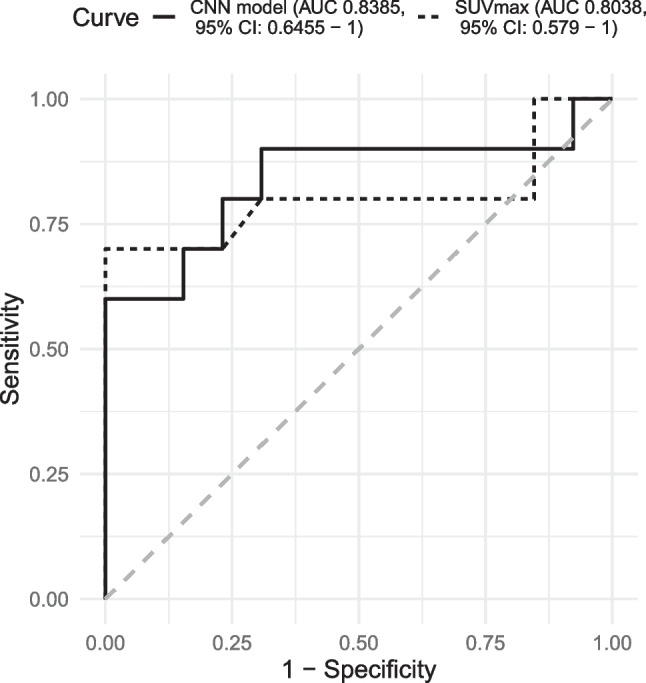
Comparison of the ROC curve between of the final CNN model and the SUVmax on the test set

The final model (Stacked 3D CNN model) obtained an area under the ROC curve of 0.8385 (95% CI: 0.6455–1.0000) on the test set (Fig. 4).

For a decision threshold (0.5039) which maximises the Youden index in the cross-validation, the model obtained a sensibility of 40.0%, a specificity of 100.0% and an accuracy of 73.9% for classifying pulmonary nodules, on the test set. Whereas, for a decision threshold (0.3149) that ensures a minimum sensitivity of 95% on the cross-validation while maximises the specificity, the model had a sensibility of 80.0%, a specificity of 69.2% and an accuracy of 73.9% on the test set.

### Comparison Between the Final CNN Model and the SUVmax

Figure 4 shows a comparison of the ROC curves between SUVmax and final CNN model. Since the ROC curves cross each other at various points, a paired comparison with the Venkatraman and Begg test [69] was applied to evaluate the equivalency of the curves rather than the area under the curve. The test statistic (E) was 22, and the two-side *P*-value was 0.7995, based on 2000 permutations.

### Grad-CAM Analysis

**Fig. 5:**
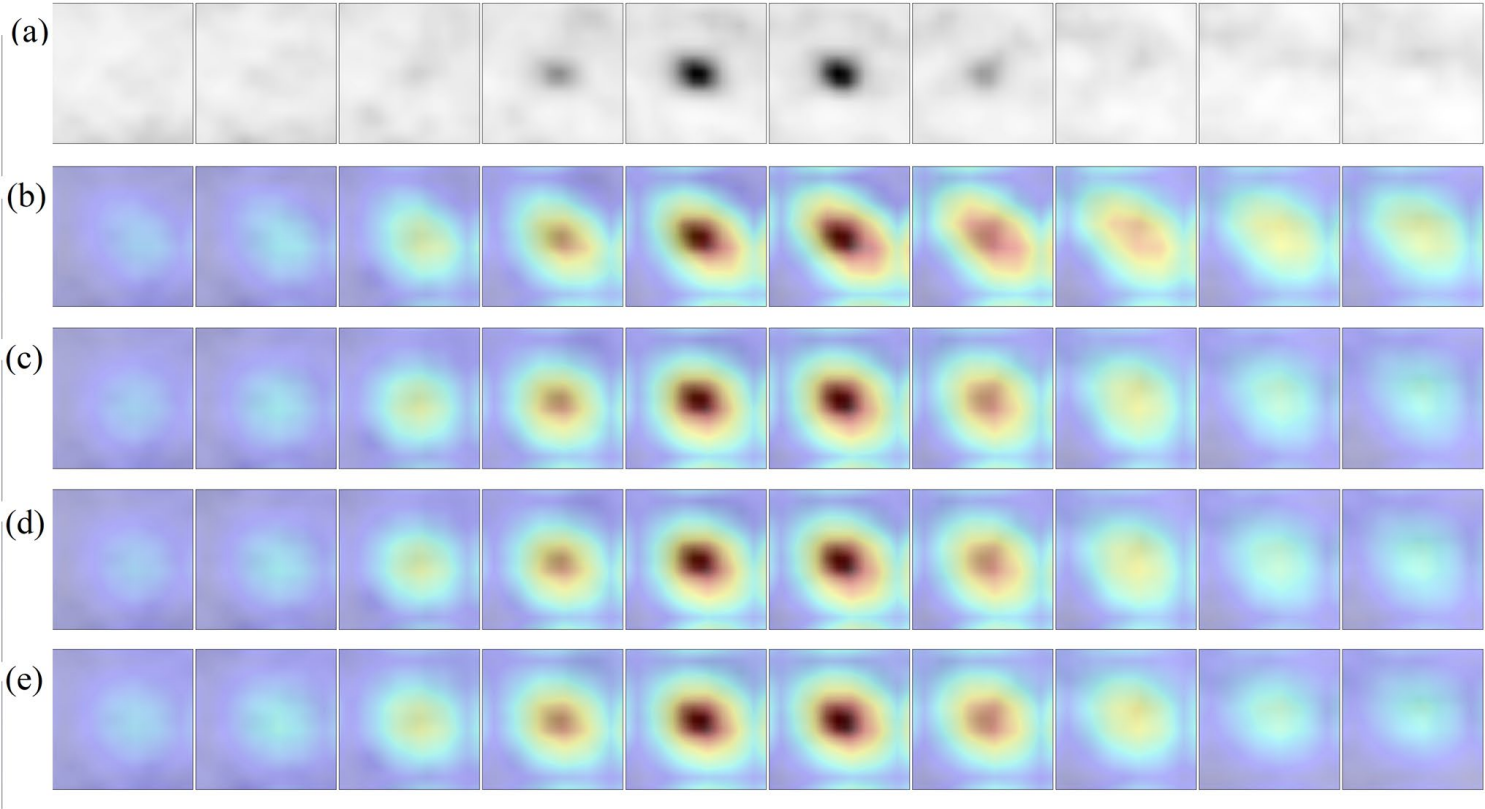
Grad-CAM 3D heatmaps generated for an input PET volume from the test set, containing a pulmonary nodule which was correctly classified as malignant by the ensemble model (true positive). **a** Thickened axial slices from the original PET volume are shown. **b**–**e** Thickened axial slices obtained by superimposing the original PET image and the Grad-CAM 3D heatmap. Each 3D CNN model version of the ensemble model has its own Grad-CAM 3D heatmap (one heatmap per row is represented)

**Fig. 6:**
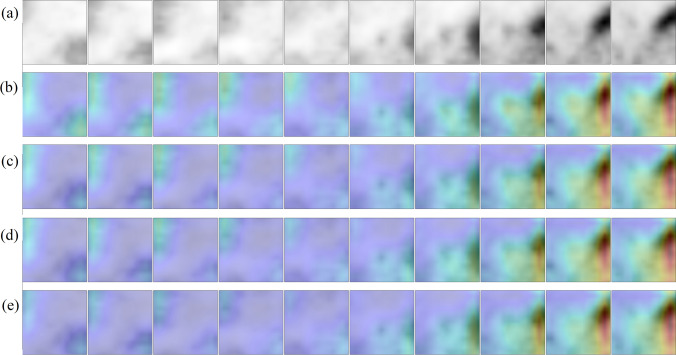
Grad-CAM 3D heatmaps generated for an input PET volume from the test set, containing a pulmonary nodule which was correctly classified as benign by the ensemble model (true negative). **a** Thickened axial slices from the original PET volume are shown. **b**–**e** Thickened axial slices obtained by superimposing the original PET image and the Grad-CAM 3D heatmap. Each 3D CNN model version of the ensemble model has its own Grad-CAM 3D heatmap (one heatmap per row is represented)

**Fig. 7:**
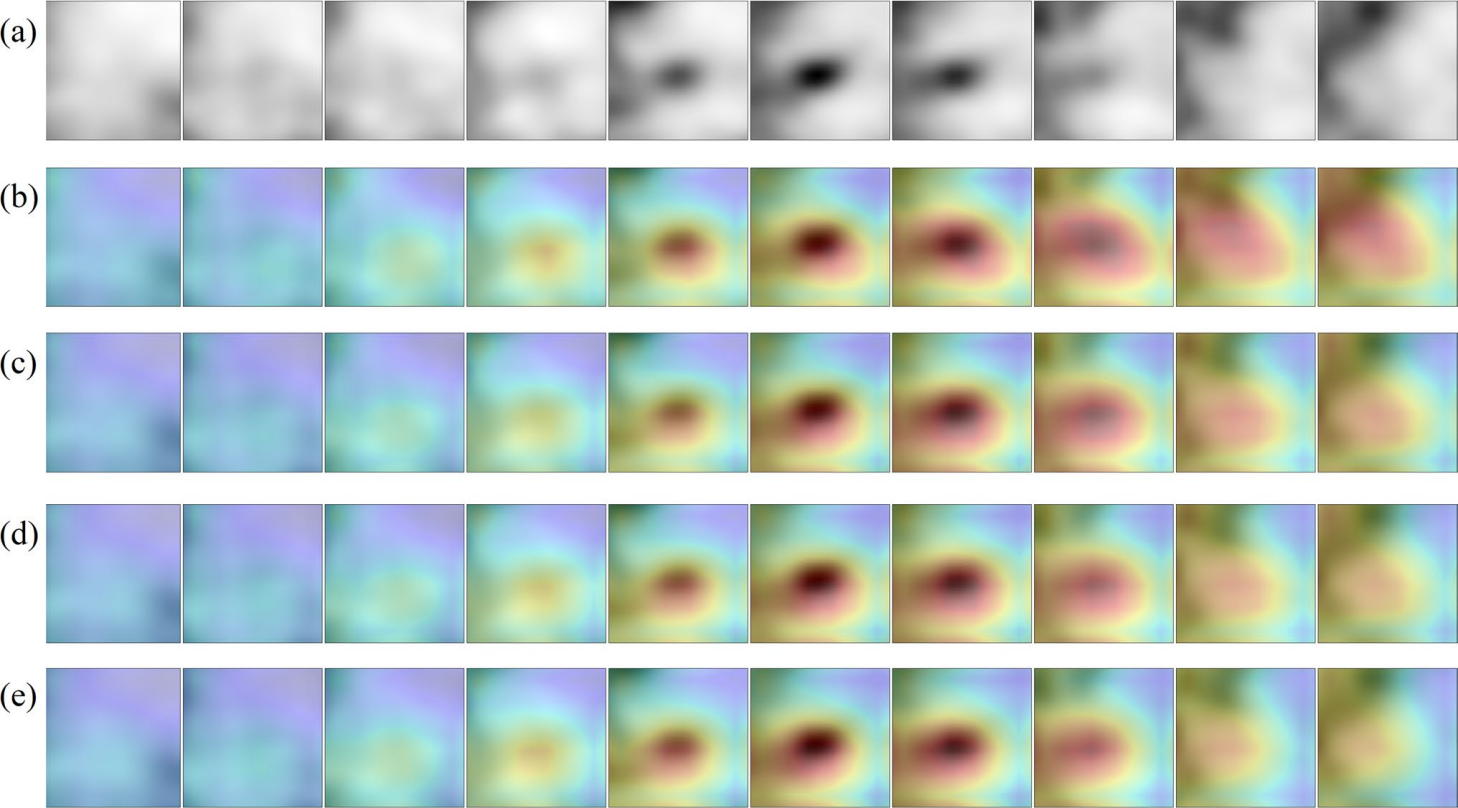
Grad-CAM 3D heatmaps generated for an input PET volume from the test set, containing a pulmonary nodule which was classified as malignant while it was benign according to the reference standard (false positive). **a** Thickened axial slices from the original PET volume are shown. **b**–**e** Thickened axial slices obtained by superimposing the original PET image and the Grad-CAM 3D heatmap. Each 3D CNN model version of the ensemble model has its own Grad-CAM 3D heatmap (one heatmap per row is represented)

**Fig. 8:**
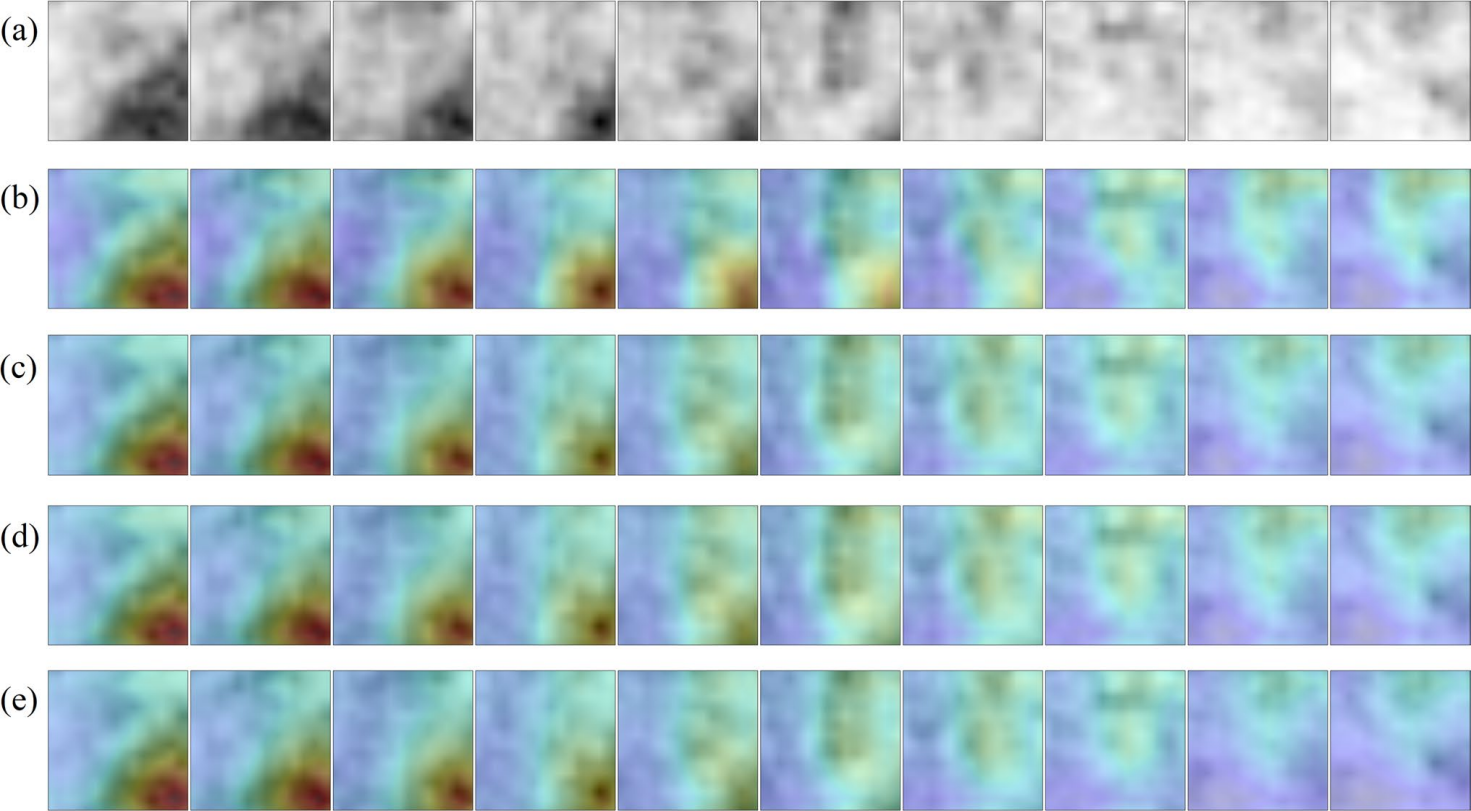
Grad-CAM 3D heatmaps generated for an input PET volume from the test set, containing a pulmonary nodule which was classified as benign while it was malignant according to the reference standard (false negative). **a** Thickened axial slices from the original PET volume are shown. **b**–**e** Thickened axial slices obtained by superimposing the original PET image and the Grad-CAM 3D heatmap. Each 3D CNN model version of the ensemble model has its own Grad-CAM 3D heatmap (one heatmap per row is represented)

Visual analysis of the Grad-CAM 3D heatmaps generated for the 3D CNN models versions (F1 to F4) that compose the ensemble model was performed for all examples of the test set. Representative cases were selected to illustrate how the ensemble model succeeds or fails. Figures 5 and 6 represent the Grad-CAM analysis of cases correctly classified by the model (true positive and true negative, respectively). Figure 7 represents the analysis of a false positive case, whereas Fig. 8 shows the analysis of a false negative case.

In any case, the four versions of the 3D CNN that compose the ensemble model pay attention to quite similar regions of the PET volume. Regarding the true positive cases, the most class-specific region includes the focal 2-[^18^F]FDG uptake in the nodule at the center of the volume and a rim of background that surrounds the nodule. In true negative cases, the 2-[^18^F]FDG uptake tends to be absent in the nodule, as such the model either attaches importance to regions close to the volume boundary (organs with physiological uptake in some cases), or to a region with non-nodular shape that includes the center of the volume. In most of false positive cases, the Grad-CAM heatmap has the highest score in an ellipsoid region at the center of volume and resembles that of the true positive cases. It coincides with a 2-[18F]FDG uptake of variable intensity in the nodule. Similarly, the Grad-CAM heatmap of false negative cases resembles that of the true negative cases, whereas the nodule has a slight or absent 2-[18F]FDG uptake.

## Discussion

We present a 3D CNN model for the classification of solid pulmonary nodules from an annotated dataset of PET images specifically created for that purpose. This classification task aimed to differentiate between benign and malignant nodules. To the best of our knowledge, this is the first study that addresses building a deep learning model for classification of indeterminate solid pulmonary nodules, using PET images as inputs.

The only attempts of using machine learning models for differential diagnosis of indeterminate pulmonary nodules have addressed classical methods and handcrafted features, namely radiomic features extracted from PET images [9, 11–20]. Despite some claims about the superiority of radiomic models over the visual interpretation or the SUVmax, the studies published to date have methodological issues that prevent a definitive conclusion about the added value of radiomics. Risk of data leakage and consequent overfitting was found in the studies of Palumbo B et al. [13], Albano B et al. [14], Ren C et al. [17] and Chen S et al. [11] because of performing exploratory analysis/feature selection in the entire dataset or the absence of a disjoint test set. Additionally, Palumbo B et al. [13], Albano B et al. [14] and Salihoğlu YS et al. [16] in their studies made a comparison of the performance metric between the radiomics model and the basal model/conventional method without performing a statistical hypothesis test, which prevents to make any inference beyond the respective dataset. The studies of Ren C et al. [17], Chen S et al. [11] and Zhang J et al. [12] made multiple comparisons of different models without controlling the family-wise error rate. Zhang J et al. [12] and Ren C et al. [17] found a superiority of the area under the ROC curve of the radiomic model regarding the SUVmax at the expense of the unnecessary and inappropriate binarization of the latter with a pre-specified threshold, which likely underestimate the area under the ROC curve of the SUVmax.

Regarding deep learning, Yong Han et al. [71] trained several classical machine learning models and a 2D CNN pre-trained (VGG-16) for distinguishing the histological subtype of pulmonary lesions in patients already diagnosed with a lung cancer, from a dataset of 1419 PET/CT fusion images. The deep learning model obtained an area under the ROC curve of 0.903. Despite the use of a deep learning algorithm, the classification problem is not the same as in the current research because it only included malignant lesions and the CT image data were also used.

The final model of the current research yielded an area under the ROC curve of 0.8385 (95% CI: 0.6455–1.000) on the test set. It has four 3D convolutional layers, three 3D max-pooling layers and three fully connected layers. It has a simpler and shallower architecture than the more recent types of networks published [72]. Since the inputs of the 3D CNN are volumes, it learns 3D spatial representations of the whole nodule, unlike a 2D CNN that receives only some slices intersecting the nodule, leading to a loss of spatial information (at least the simplest approaches) [73–75]. For this reason, a 3D CNN was preferred. However, a 3D CNN has the cost of a higher number of parameters and higher risk of overfitting [73]. As such, the capacity of the model was carefully adjusted to the problem and size of the dataset. Several regularisation methods were also applied, such as early stopping and L2 regularisation.

The probabilistic predictions were converted to target classes by determining an optimum threshold. Two approaches were used. The Youden’s index and a pre-assigned value for the sensitivity both yielded an accuracy of 73.91% in the test set. However, the sensitivity obtained with the second method in the test set was much more favourable (80% vs. 40%). This is explained by the characteristics of each method and by the variance associated to the reduced size of the test set (23 images). The specificity of the second method of threshold moving was 73.91%. A threshold that maximises the specificity, setting a minimum value sensitivity of 95% (derived from the cross-validation), can be a more appropriate approach for the current problem because a greater cost is placed on false negatives than on false positives, being assumed that the cost of missing a malignant lesion is higher than the cost of additional investigations and psychological distress caused by a false positive.

As a secondary endpoint, the performance of the 3D CNN model was compared with the SUVmax of the nodules. The model had an area under the ROC curve higher than the SUVmax in the test set (0.8385 vs. 0.8038). However, the equivalency between the two ROC curves was not rejected by a hypothesis test that compared their shape. Because the test set was not sized to ensure an adequate statistical power to the applied test, this negative result requires confirmation in well-powered studies.

Other types of 3D CNNs were also proposed, achieving a slightly lower area under the ROC curve than the Stacked 3D CNN in the cross-validation. These networks were inspired by VGG-16 [57] and Inception-v2 [58]. They are deeper and have some features that make them more efficient, such as factorisation of convolutions, introduction of the sparsity in the network or 1 × 1 × 1 convolutions.

Deep learning models usually need to be trained in a big dataset to prevent overfitting [76]. However, building an annotated dataset in medical imaging is a time-consuming and a labour-intensive task. Furthermore, the particularity of the task and the imaging modality involved imply that the number of images available may be limited, as in the current research. Even though, a model was successfully trained and regularised.

Models trained with transfer learning had a lower classification performance in the cross-validation than those models trained with random weight initialisation. This could be explained by the difference between the source domain where the CNN was pre-trained (ImageNet) and the target domain, by the 2D architecture requiring 2D inputs, or by the type of pre-trained network (ResNet). Models trained with data augmentation also had a classification performance in the cross-validation consistently lower than those trained in the original training dataset. It was out scope of the phase of model selection to make statistical inference from the differences between the models, so it is unknown the meaning of those differences as well as their cause. It is hypothesised that the size of original dataset was insufficient for the augmentations to produce any effect, or the type or the parameters of the transformations were not the most appropriate to lead to an improvement of performance in this specific type of image data and problem, or the factor of augmentation was insufficient. High-quality and representative datasets are essential for developing machine learning models and for ensuring they have acceptable generalisation performance on unseen cases. Although this is a retrospective study, the eligible population was explicitly and accurately defined. The quality of a dataset also depends on the quality of the reference standard. Predictive modelling for diagnosis purposes follows the same principles as the diagnosis tests regarding obtaining an unbiased reference standard [77]. A proof about the presence or absence of the target disease should be obtained without knowledge of the index test and vice versa [77, 78]. Similarly, the reference standard should not contain information from the data where a predictive model will be built; otherwise, the model will have an optimistic performance [77]. In the current study, that incorporation bias was prevented by using the result of the histopathological or cytopathological examination of a specimen obtained by biopsy or surgical excision or, alternatively, a follow-up period with CT. Therefore, there was a differential verification of the disease status. The histopathological characterisation of the lesion was the main method to obtain the reference standard, representing 62.8% of the cases. The CT imaging follow-up was the method to obtain the definitive diagnosis in the remaining cases (except one), with 85% of the patients having a follow-up time of at least 2 years. Surgical resection is the gold standard for definitive diagnosis of pulmonary nodules [79] that is an unbiased reference standard. The biopsy also provides direct evidence of malignancy, but there is a risk of non-specific benign changes as false negatives [80]. To eliminate that risk of bias in the biopsy, only definitive evidence of a benign pathology was considered (on first or repeated biopsies); otherwise, the follow-up criterion was applied. Imaging follow-up provides an indirect, but still strong, evidence of the status of the nodule, leading to a low risk of bias in the ground-truth. The defined follow-up criteria ensured that a malignant tumour is missed in <1% of cases, according to the previous literature [8].

In malignant nodules, Grad-CAM analysis showed that the model tends to pay attention to the nodule region during the decision, whereas in benign nodules, either no object in the lung receives particular attention or a central region with non-nodular shape receives attention. Moreover, the size and the shape of the most class-discriminant region seem to assume importance for the model decision, which raises the hypothesis that the decision can rely on nodule-background contrast and on the metabolic shape of the nodule. Model failures are explained by the similarity between the Grad-CAM heatmap of a given image and those of the misidentified class.

This study has some limitations. The model was built in a relatively small dataset. Despite the efforts of regularisation, its performance in a larger dataset is unknown. Also, the test set was small, so the generalisation performance is highly dependent on the data split. It is unknown how the model generalises in a PET scanner basis, including with images obtained from other PET scanner types not used in the current dataset.

Because this is a retrospective study, the decision of performing a PET/CT exam or a biopsy or excision of the pulmonary nodule, as well as the duration of follow-up period, was at the discretion of the attending physician. The decision criteria may have changed over time, as part of the evolution of knowledge in this area, and according to the attending physician, resulting in selection and partial verification biases [81]. When multiple nodules were present, the dataset only included the most suspicious nodule from each patient, instead of all the nodules, but in practice it is important to know the status of all of them.

The image data stores standardised uptake value (SUV) by voxel. SUV has been popularised, but another less used measure was claimed to be more accurate: the standardised uptake value normalised by the lean body mass (SUL) [82], once the lean and the fat tissues have different metabolic profiles. Image data were not recalculated to show SUL because the DICOM files from one of the PET scanners did not have the height data recorded.

As future work, we suggest evaluating the proposed model in a larger dataset, preferably collected prospectively from multiple centres and PET/CT scanners, and possibly to retrain it in those data. Another proposal is to train a CNN model that considers not only the PET data, but also the low-dose CT data from the same exam and non-imaging features.

The task in the current research required manual nodule location before the automatic classification. However, nodule location and classification can be combined in a single machine learning task (nodule detection).

## Conclusion

In this study, we developed a 3D CNN model for automatic classification of indeterminate solid pulmonary nodules from an annotated dataset of 2-[^18^F]FDG PET images which was specifically created for that purpose. The model was effective for differentiating malignant and benign nodules and has potential for improving the differential diagnosis of pulmonary nodules.

### Supplementary Information

ESM 1 (PDF 564 kb)

## Data Availability

The datasets generated during and/or analysed during the current study are available from the corresponding author on reasonable request.

## Footnotes

1 https://developer.nvidia.com/gtc/2019/video/s9911

## References

[1] Sung H, Ferlay J, Siegel RL, Laversanne M, Soerjomataram I, Jemal A (2021). Global Cancer Statistics 2020: GLOBOCAN estimates of incidence and mortality worldwide for 36 cancers in 185 countries. CA: A Cancer J Clin..

[2] Qian F, Yang W, Chen Q, Zhang X, Han B (2018). Screening for early stage lung cancer and its correlation with lung nodule detection. J Thoracic Dis..

[3] Woodard GA, Jones KD, Jablons DM, Reckamp KL (2016). Lung cancer staging and prognosis. Lung cancer: treatment and research.

[4] Elia S, Loprete S, De Stefano A, Hardavella G (2019). Does aggressive management of solitary pulmonary nodules pay off?. Breathe..

[5] Ruparel M, Quaife SL, Navani N, Wardle J, Janes SM, Baldwin DR (2016). Pulmonary nodules and CT screening: the past, present and future. Thorax..

[6] The National Lung Screening Trial Research Team (2011). Reduced lung-cancer mortality with low-dose computed tomographic screening. N Engl J Med..

[7] MacMahon H, Naidich DP, Goo JM, Lee KS, Leung ANC, Mayo JR (2017). Guidelines for management of incidental pulmonary nodules detected on CT images: from the Fleischner Society 2017. Radiology..

[8] Callister MEJ, Baldwin DR, Akram AR, Barnard S, Cane P, Draffan J (2015). British Thoracic Society guidelines for the investigation and management of pulmonary nodules: accredited by NICE. Thorax.

[9] Herder GJ, van Tinteren H, Golding RP, Kostense PJ, Comans EF, Smit EF (2005). Clinical prediction model to characterize pulmonary nodules: validation and added value of 18 F-fluorodeoxyglucose positron emission tomography. Chest..

[10] Ruilong Z, Daohai X, Li G, Xiaohong W, Chunjie W, Lei T (2017). Diagnostic value of 18F-FDG-PET/CT for the evaluation of solitary pulmonary nodules: a systematic review and meta-analysis. Nucl Med Commun..

[11] Chen S, Harmon S, Perk T, Li X, Chen M, Li Y (2017). Diagnostic classification of solitary pulmonary nodules using dual time 18F-FDG PET/CT image texture features in granuloma-endemic regions. Sci Rep..

[12] Zhang J, Ma G, Cheng J, Song S, Zhang Y, Shi LQ (2020). Diagnostic classification of solitary pulmonary nodules using support vector machine model based on 2-[18F]fluoro-2-deoxy-D-glucose PET/computed tomography texture features. Nucl Med Commun..

[13] Palumbo B, Bianconi F, Palumbo I, Fravolini ML, Minestrini M, Nuvoli S (2020). Value of shape and texture features from 18F-FDG PET/CT to discriminate between benign and malignant solitary pulmonary nodules: an experimental evaluation. Diagnostics..

[14] Albano D, Gatta R, Marini M, Rodella C, Camoni L, Dondi F (2021). Role of 18F-FDG PET/CT radiomics features in the differential diagnosis of solitary pulmonary nodules: diagnostic accuracy and comparison between two different pet/ct scanners. J Clin Med..

[15] Niu R, Gao J, Shao X, Wang J, Jiang Z, Shi Y (2021). Maximum standardized uptake value of 18F-deoxyglucose PET imaging increases the effectiveness of CT radiomics in differentiating benign and malignant pulmonary ground-glass nodules. Front Oncol..

[16] Salihoğlu YS, Erdemir RU, Püren BA, Özdemir S, Uyulan Ç, Ergüzel TT (2022). Diagnostic performance of machine learning models based on 18 F-FDG PET/CT radiomic features in the classification of solitary pulmonary nodules. Mol Imaging Radionucl Ther..

[17] Ren C, Xu M, Zhang J, Zhang F, Song S, Sun Y, et al. Classification of solid pulmonary nodules using a machine-learning nomogram based on 18F-FDG PET/CT radiomics integrated clinicobiological features. Ann Trans Med. 2022;10 10.21037/atm-22-2647. 10.21037/atm-22-2647 PMC9816842 36618813

[18] Teramoto A, Tsujimoto M, Inoue T, Tsukamoto T, Imaizumi K, Toyama H (2019). Automated classification of pulmonary nodules through a retrospective analysis of conventional CT and two-phase PET images in patients undergoing biopsy. Asia Ocean J Nucl Med Biol..

[19] Guo HY, Lin JT, Huang HH, Gao Y, Yan MR, Sun M (2020). Development and validation of a 18F-FDG PET/CT-based clinical prediction model for estimating malignancy in solid pulmonary nodules based on a population with high prevalence of malignancy. Clin Lung Cancer..

[20] Wang L, Chen Y, Tang K, Lin J, Zhang H (2018). The value of 18 F-FDG PET/CT mathematical prediction model in diagnosis of solitary pulmonary nodules. BioMed Res Int..

[21] Zwanenburg A, Vallières M, Abdalah MA, Aerts HJWL, Andrearczyk V, Apte A (2020). The image biomarker standardization initiative: standardized quantitative radiomics for high-throughput image-based phenotyping. Radiology..

[22] Zwanenburg A (2019). Radiomics in nuclear medicine: robustness, reproducibility, standardization, and how to avoid data analysis traps and replication crisis. Eur J Nucl Med Mol Imaging..

[23] Pfaehler E, Zhovannik I, Wei L, Boellaard R, Dekker A, Monshouwer R (2021). A systematic review and quality of reporting checklist for repeatability and reproducibility of radiomic features. Phys Imaging Radiat Oncol..

[24] LeCun Y, Bengio Y, Hinton G (2015). Deep learning. Nature..

[25] Esteva A, Kuprel B, Novoa RA, Ko J, Swetter SM, Blau HM (2017). Dermatologist-level classification of skin cancer with deep neural networks. Nature..

[26] Brinker TJ, Hekler A, Enk AH, Berking C, Haferkamp S, Hauschild A (2019). Deep neural networks are superior to dermatologists in melanoma image classification. Europ J Cancer..

[27] Yang Y, Wang J, Xie F, Liu J, Shu C, Wang Y (2021). A convolutional neural network trained with dermoscopic images of psoriasis performed on par with 230 dermatologists. Comput Biol Med..

[28] Kermany DS, Goldbaum M, Cai W, Valentim CCS, Liang H, Baxter SL (2018). Identifying medical diagnoses and treatable diseases by image-based deep learning. Cell.

[29] Tan TE, Anees A, Chen C, Li S, Xu X, Li Z (2021). Retinal photograph-based deep learning algorithms for myopia and a blockchain platform to facilitate artificial intelligence medical research: a retrospective multicohort study. Lancet Digital Health..

[30] De Fauw J, Ledsam JR, Romera-Paredes B, Nikolov S, Tomasev N, Blackwell S (2018). Clinically applicable deep learning for diagnosis and referral in retinal disease. Nat Med..

[31] Lin D, Xiong J, Liu C, Zhao L, Li Z, Yu S (2021). Application of comprehensive artificial intelligence retinal expert (CARE) system: a national real-world evidence study. Lancet Digital Health..

[32] Ehteshami Bejnordi B, Veta M (2017). Johannes van Diest P, van Ginneken B, Karssemeijer N, Litjens G, et al. Diagnostic assessment of deep learning algorithms for detection of lymph node metastases in women with breast cancer. JAMA..

[33] Yu G, Sun K, Xu C, Shi XH, Wu C, Xie T (2021). Accurate recognition of colorectal cancer with semi-supervised deep learning on pathological images. Nat Commun..

[34] Huang B, Tian S, Zhan N, Ma J, Huang Z, Zhang C (2021). Accurate diagnosis and prognosis prediction of gastric cancer using deep learning on digital pathological images: a retrospective multicentre study. eBioMedicine..

[35] Rajpurkar P, Irvin J, Ball RL, Zhu K, Yang B, Mehta H (2018). Deep learning for chest radiograph diagnosis: a retrospective comparison of the CheXNeXt algorithm to practicing radiologists. PLOS Med..

[36] Lin A, Manral N, McElhinney P, Killekar A, Matsumoto H, Kwiecinski J (2022). Deep learning-enabled coronary CT angiography for plaque and stenosis quantification and cardiac risk prediction: an international multicentre study. Lancet Digital Health..

[37] Ardila D, Kiraly AP, Bharadwaj S, Choi B, Reicher JJ, Peng L (2019). End-to-end lung cancer screening with three-dimensional deep learning on low-dose chest computed tomography. Nat Med..

[38] Wang G, Liu X, Shen J, Wang C, Li Z, Ye L (2021). A deep-learning pipeline for the diagnosis and discrimination of viral, non-viral and COVID-19 pneumonia from chest X-ray images. Nat Biomed Eng..

[39] Fedorov A, Beichel R, Kalpathy-Cramer J, Finet J, Fillion-Robin JC, Pujol S (2012). 3D Slicer as an image computing platform for the quantitative imaging network. Magnet Reson Imaging..

[40] Vapnik VN (1999). An overview of statistical learning theory. IEEE Trans Neural Netw..

[41] Wang Q, Ma Y, Zhao K, Tian Y (2022). A comprehensive survey of loss functions in machine learning. Ann Data Sci..

[42] Goodfellow I, Bengio Y, Courville A (2016). Deep Learning.

[43] Kohavi R (1995). A study of cross-validation and bootstrap for accuracy estimation and model selection. Proceedings of the 14th International Joint Conference on Artificial Intelligence - Volume 2.

[44] Chollet F, Allaire J (2018). Deep Learning with R.

[45] Shorten C, Khoshgoftaar TM (2019). A survey on image data augmentation for deep learning. J Big Data..

[46] Comninos P (2006). Three-dimensional transformations. in mathematical and computer programming techniques for computer graphics.

[47] Teymurazyan A, Riauka T, Jans HS, Robinson D (2013). Properties of noise in positron emission tomography images reconstructed with filtered-backprojection and row-action maximum likelihood algorithm. J Digital Imaging..

[48] R Core Team (2019). R: A Language and Environment for Statistical Computing.

[49] Allaire JJ, Tang Y, Tensorflow: R Interface to ‘TensorFlow’ (2019). R package version 2.0.0.

[50] Allaire JJ, Chollet F. keras: R Interface to ‘Keras’. R package version 2.2.5.0. The R Foundation for Statistical Computing; 2019.

[51] Ushey K, Allaire JJ, Tang Y. reticulate: Interface to ‘Python’. The R Foundation for Statistical Computing; 2020.

[52] Abadi M, Agarwal A, Barham P, Brevdo E, Chen Z, Citro C, et al. TensorFlow: large-scale machine learning on heterogeneous systems. 2015. Software available from tensorflow.org.

[53] Van Rossum G, Drake FL (2009). Python 3 Reference Manual.

[54] Kingma DP, Ba J, Bengio Y, Le Cun Y (2015). Adam: A method for stochastic optimization. 3rd International Conference on Learning Representations.

[55] Mahsereci M, Balles L, Lassner C, Hennig P. Early Stopping without a validation set. CoRR. 2017; abs/1703.09580

[56] Krizhevsky A, Sutskever I, Hinton GE, Pereira F, Burges CJ, Bottou L, Weinberger KQ (2012). ImageNet classification with deep convolutional neural networks. Advances in Neural Information Processing Systems.

[57] Simonyan K, Zisserman A. Very deep convolutional networks for large-scale image recognition. In: Bengio Y, Le Cun Y, editors. 3rd International Conference on Learning Representations, ICLR. San Diego, CA, USA; 2015. https://ora.ox.ac.uk/objects/uuid:60713f18-a6d1-4d97-8f45-b60ad8aebbce. Accessed 1 Dec 2022.

[58] Szegedy C, Vanhoucke V, Ioffe S, Shlens J, Wojna Z (2015). Rethinking the inception architecture for computer vision.

[59] Gu J, Wang Z, Kuen J, Ma L, Shahroudy A, Shuai B (2018). Recent advances in convolutional neural networks. Pattern Recognit..

[60] Lu YY, Em KG (2020). Dying ReLU and initialization: theory and numerical examples. Commun Comput Phys..

[61] He K, Zhang X, Ren S, Sun J (2015). Delving deep into rectifiers: surpassing human-level performance on ImageNet classification.

[62] Weiss K, Khoshgoftaar TM, Wang D (2016). A survey of transfer learning. J Big Data..

[63] Deng J, Dong W, Socher R, Li LJ, Li K, Fei-Fei L (2009). Imagenet: A large-scale hierarchical image database.

[64] He K, Zhang X, Ren S, Sun J. Deep residual learning for image recognition. 2016 IEEE Conference on Computer Vision and Pattern Recognition (CVPR); 2016. pp. 770–8.

[65] Srivastava N, Hinton G, Krizhevsky A, Sutskever I, Salakhutdinov R (2014). Dropout: a simple way to prevent neural networks from overfitting. J Mach Learn Res..

[66] Robin X, Turck N, Hainard A, Tiberti N, Lisacek F, Sanchez JC (2011). pROC: an open-source package for R and S+ to analyze and compare ROC curves. BMC Bioinform..

[67] DeLong ER, DeLong DM, Clarke-Pearson DL (1988). Comparing the areas under two or more correlated receiver operating characteristic curves: a nonparametric approach. Biometrics..

[68] López-Ratón M, Rodríguez-Álvarez MX, Cadarso-Suárez C, Gude-Sampedro F (2014). OptimalCutpoints: an R package for selecting optimal cutpoints in diagnostic tests. J Stat Software..

[69] Venkatraman ES, Begg CB (1996). A distribution-free procedure for comparing receiver operating characteristic curves from a paired experiment. Biometrika..

[70] Selvaraju RR, Cogswell M, Das A, Vedantam R, Parikh D, Batra D (2017). Grad-CAM: Visual Explanations from Deep Networks via Gradient-Based Localization.

[71] Han Y, Ma Y, Wu Z, Zhang F, Zheng D, Liu X (2021). Histologic subtype classification of non-small cell lung cancer using PET/CT images. Eur J Nucl Med Mol Imaging..

[72] Khan A, Sohail A, Zahoora U, Qureshi AS (2020). A survey of the recent architectures of deep convolutional neural networks. Artif Intell Rev..

[73] Hu J, Kuang Y, Liao B, Cao L, Dong S, Li P (2019). A multichannel 2D convolutional neural network model for task-evoked fMRI data classification. Comput Intell Neurosci..

[74] Yu Q, Xia Y, Xie L, Fishman EK, Yuille AL (2019). Thickened 2D networks for 3D medical image segmentation.

[75] Liu M, Cheng D, Yan W, ADNI. Classification of Alzheimer’s disease by combination of convolutional and recurrent neural networks using FDG-PET images. Front Neuroinform. 2018;12 10.3389/fninf.2018.00035. 10.3389/fninf.2018.00035 PMC6018166 29970996

[76] Kukačka J, Golkov V, Cremers D (2018). Regularization for deep learning: a taxonomy. In: 6th International Conference on Learning Representations.

[77] Moons KGM, de Groot JAH, Bouwmeester W, Vergouwe Y, Mallett S, Altman DG (2014). Critical appraisal and data extraction for systematic reviews of prediction modelling studies: the CHARMS checklist. PLOS Med..

[78] Weinstein S, Obuchowski NA, Lieber ML (2005). Clinical evaluation of diagnostic tests. Am J Roentgenol..

[79] Ricciardi S, Davini F, Manca G, De Liperi A, Romano G, Zirafa CC (2020). Radioguided surgery, a cost-effective strategy for treating solitary pulmonary nodules: 20-year experience of a single center. Clin Lung Cancer..

[80] Laurent F, Montaudon M, Latrabe V, Bégueret H (2003). Percutaneous biopsy in lung cancer. Eur J Radiol..

[81] Schmidt RL, Factor RE (2013). Understanding sources of bias in diagnostic accuracy studies. Arch Pathol Lab Med..

[82] Joo Hyun O, Lodge MA, Wahl RL (2016). Practical PERCIST: a simplified guide to PET response criteria in solid tumors 1.0. Radiology.

