# Supplementary material for: Classification of Pulmonary Nodules in 2-[18F]FDG PET/CT Images with a 3D Convolutional Neural Network
Source: Nucl Med Mol Imaging. 2023 Aug 30;58(1):9–24. doi: 10.1007/s13139-023-00821-6 (PMC10796312; doi:10.1007/s13139-023-00821-6)
Supplement: Supplementary file 1 — (PDF 564 kb) [file 13139_2023_821_MOESM1_ESM.pdf]

Classification of pulmonary nodules in 2-[^{18}F]FDG PET/CT images with a 3D convolutional neural network

Victor Manuel Alves[1,2*], Jaime dos Santos Cardoso[3,4] and João Gama[1,4]

[1]Faculty of Economics, University of Porto, Rua Dr. Roberto Frias, Porto, 4200-464 Porto, Portugal.
[2*]Department of Nuclear Medicine, University Hospital Center of São João, Alameda Prof. Hernâni Monteiro, Porto, 4200–319, Portugal.
[3]Faculty of Engineering, University of Porto, Rua Dr. Roberto Frias, Porto, 4200-465, Portugal.
[4]Institute for Systems and Computer Engineering, Technology and Science (INESC TEC), Rua Dr. Roberto Frias, Porto, 4200-465, Portugal.

*Corresponding author(s). E-mail(s): alves.vm@pm.me; Contributing authors: jaime.cardoso@fe.up.pt; jgama@fep.up.pt;

A Supporting Material

A.1 Supplementary Methods

A.1.1 Efficiency improvement of a 3D CNN regarding a 2D CNN - the case of VGG-like network

Factorisation of convolutions imposes a greater reduction in the number of parameters in a 3D than in a 2D network as explained next. Assuming an equal number of input and output characteristic maps, represented by m and ignoring the number of biases, for a given block of n stacked $k \times k \times k$ convolutional layers, the number of weights can be determined by $n(k^3m^2)$ Simonyan and Zisserman (2015). So, a stack of two $3 \times 3 \times 3$ convolutional layers has

$2(3^3m^2) = 54m^2$ weights, whereas the equivalent single $5 \times 5 \times 5$ convolutional layer would have $5^3m^2 = 125m^2$, which represents a reduction of 57% of the number of weights. A similar implementation in a 2D CNN only imposes a reduction of 28% of the number of weights. Replacing a $7 \times 7 \times 7$ convolutional layer by the equivalent stack of three $3 \times 3 \times 3$ convolutional layers leads to a reduction of the number of weights in 76%, whereas in a 2D CNN, that would lead to a reduction of 45%.

A.1.2 Representation of VGG-like network

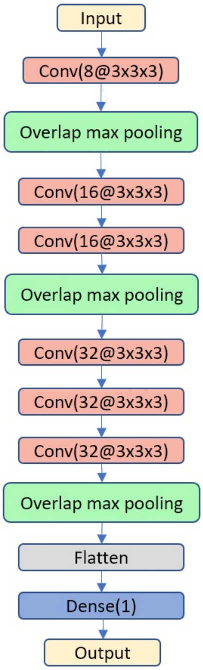

Fig. A1: Network architecture of VGG-like network.

A.1.3 Representation of Inception-v2-like network

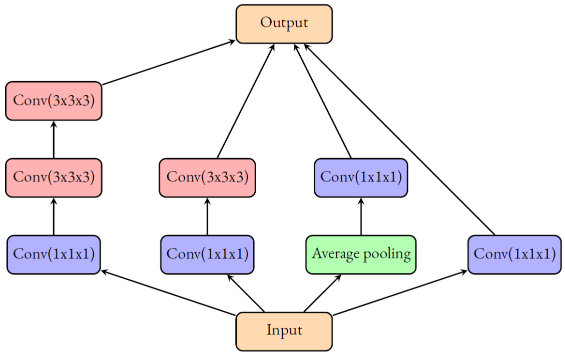

Fig. A2: 3D Inception module 1.

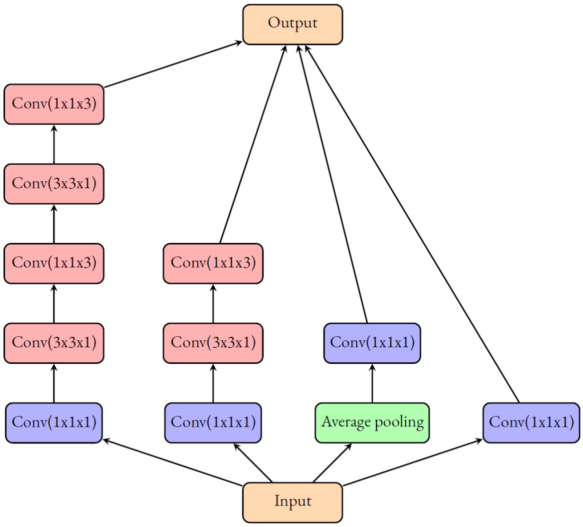

Fig. A3: 3D Inception module 2.

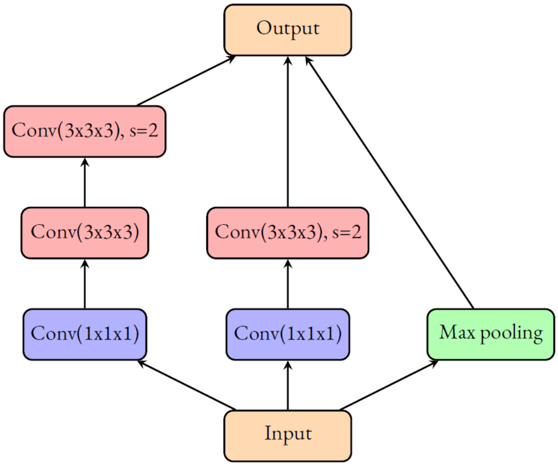

Fig. A4: 3D Inception reduction module 1; s=2 means strides of [2, 2, 2]

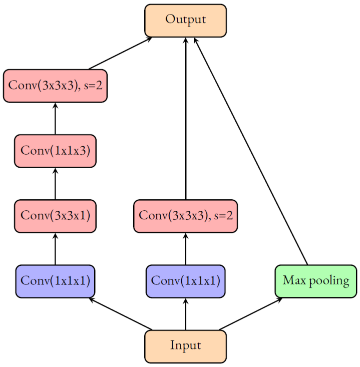

Fig. A5: 3D Inception reduction module 2; s=2 means strides of [2, 2, 2]

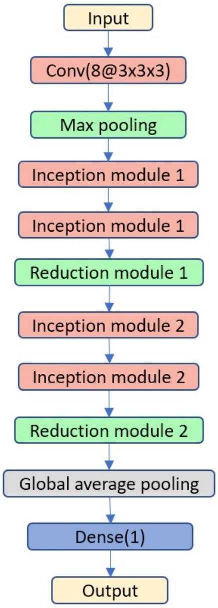

Fig. A6: Inception-v2-like network

A.2 Supplementary Results

Table A1: Area under the ROC curve of the best stacked 3D model over 10 iterations of cross-validation.

	Area under the ROC curve					
Iteration	F1	F2	F3	F4	Mean	SD[1]
1	0.7917	0.9000	0.8583	0.9790	0.8823	0.0784
2	0.7917	0.9000	0.8750	0.9790	0.8864	0.0772
3	0.7917	0.9000	0.8750	0.9650	0.8829	0.0717
4	0.7917	0.9000	0.8750	0.9650	0.8829	0.0717
5	0.7917	0.9000	0.8583	0.9720	0.8805	0.0756
6	0.7917	0.9000	0.8667	0.9720	0.8826	0.0749
7	0.7917	0.9000	0.8667	0.9720	0.8826	0.0749
8	0.7917	0.9000	0.8750	0.9720	0.8847	0.0744
9	0.7917	0.9000	0.8500	0.9720	0.8784	0.0765
10	0.7917	0.9000	0.8583	0.9650	0.8788	0.0728
**Mean**	**0.7917**	**0.9000**	**0.8658**	**0.9713**	**0.8822**	**0.0748**

[1]SD: standard deviation

Table A2: Area under the ROC curve of the best VGG-like model over 10 iterations of cross-validation.

	Area under the ROC curve					
Iteration	F1	F2	F3	F4	Mean	SD[1]
1	0.7333	0.9250	0.9167	0.9371	0.8780	0.0968
2	0.7333	0.9250	0.9417	0.9091	0.8773	0.0969
3	0.7333	0.9250	0.9333	0.9231	0.8787	0.0970
4	0.7333	0.9250	0.9333	0.9301	0.8804	0.0981
5	0.7333	0.9250	0.9333	0.9161	0.8769	0.0960
6	0.7333	0.9250	0.925	0.8881	0.8679	0.0914
7	0.7333	0.9333	0.9167	0.9301	0.8784	0.0969
8	0.7333	0.9250	0.9083	0.9301	0.8742	0.0944
9	0.7333	0.9167	0.9333	0.9021	0.8714	0.0929
10	0.7333	0.9250	0.9167	0.9301	0.8763	0.0954
**Mean**	**0.7333**	**0.9250**	**0.9258**	**0.9196**	**0.8760**	**0.0956**

[1]SD: standard deviation

Table A3: Area under the ROC curve of the best Inception-v2-like model over 10 iterations of cross-validation.

	Area under the ROC curve					
Iteration	F1	F2	F3	F4	Mean	SD[1]
1	0.7250	0.9083	0.8917	0.9510	0.8690	0.0992
2	0.7250	0.9083	0.8917	0.9650	0.8725	0.1032
3	0.7250	0.9083	0.8917	0.9650	0.8725	0.1032
4	0.725	0.9083	0.8917	0.9510	0.8690	0.0992
5	0.7250	0.9083	0.8917	0.9301	0.8638	0.0938
6	0.7250	0.9083	0.8917	0.9510	0.8690	0.0992
7	0.7250	0.9083	0.8917	0.9371	0.8655	0.0955
8	0.7250	0.9083	0.8917	0.9371	0.8655	0.0955
9	0.7250	0.9083	0.8917	0.9580	0.8708	0.1012
10	0.7250	0.9083	0.8917	0.9650	0.8725	0.1032
**Mean**	**0.7250**	**0.9083**	**0.89170**	**0.9510**	**0.8690**	**0.0992**

[1]SD: standard deviation

References

Simonyan K, Zisserman A (2015) Very deep convolutional networks for large-scale image recognition. In: Bengio Y, LeCun Y (eds) 3rd International Conference on Learning Representations, ICLR 2015, San Diego, CA, USA, May 7-9, 2015, Conference Track Proceedings, URL http://arxiv.org/abs/1409.1556
